# Adult-onset Still’s disease: A disease at the crossroad of innate immunity and autoimmunity

**DOI:** 10.3389/fmed.2022.881431

**Published:** 2022-08-22

**Authors:** Shijia Rao, Lemuel Shui-Lun Tsang, Ming Zhao, Wei Shi, Qianjin Lu

**Affiliations:** ^1^Department of Dermatology, The Second Xiangya Hospital, Central South University, Hunan Key Laboratory of Medical Epigenomics, Changsha, China; ^2^College of Medicine, University of Tennessee Health Science Center, Memphis, TN, United States; ^3^Department of Dermatology, Xiangya Hospital, Central South University, Changsha, China; ^4^Institute of Dermatology, Chinese Academy of Medical Sciences and Peking Union Medical College, Nanjing, China; ^5^Key Laboratory of Basic and Translational Research on Immune-Mediated Skin Diseases, Chinese Academy of Medical Sciences, Nanjing, China; ^6^Jiangsu Key Laboratory of Molecular Biology for Skin Diseases and STIs, Nanjing, China

**Keywords:** adult-onset Still’s disease, autoinflammatory disease, autoimmunity, immunopathogenesis, biological treatment

## Abstract

Adult-onset Still’s disease (AOSD) is a rare disease affecting multiple systems and organs with unknown etiology, and the clinical symptoms are usually described as spiking fever, arthritis, evanescent salmon-pink eruptions, lymphadenopathy, splenomegaly, and other manifestations. The laboratory indicators are not specific, often presenting as increased leukocyte counts and neutrophil percentage, elevated erythrocyte sedimentation rate (ESR) and C-reactive protein (CRP), hyperferritinemia, and increased inflammatory factors. ANA, ENA, and RF are negative. According to those unspecific clinical presentations and laboratory findings, infection, tumor, connective tissue disease, and other diseases must be ruled out before diagnosis. The diagnosis of AOSD is a great challenge for clinicians. The mechanism of AOSD pathogenesis is complicated and still being studied. There is a new opinion that atypical persistent skin eruptions (APSEs) with specific histological manifestations are unique for AOSD, and APSEs might be on a spectrum with classical evanescent eruptions. Studies on APSEs showed that IL-1β and IFN-γ are strongly correlated with the pathogenesis of necrosis keratinocytes in APSEs. IL-1β is strongly involved in inflammatory disease when it is abnormal, and plays an important role in the pathogenesis of neutrophil dermatosis. In the early stage of AOSD, skin lesions appear to be evanescent urticaria-like eruptions accompanied by fever, and only neutrophils infiltrate around the blood vessels in the dermis pathologically. As the course of the disease progresses, IL-1β is gradually released. Through the stimulation of other inflammatory factors and the influence of unknown factors, IL-1β gradually infiltrates into the stratum corneum and finally accumulates around the necrotic keratinocytes of the stratum corneum. However, the detailed mechanism is still unknown. IFN-γ could play a pro-inflammatory or regulatory role in some disorders. IL-1β can enhance the expression of IFN-γ, and IFN-γ can cause keratinocyte apoptosis by activating the autocrine of caspase. Also, several pieces of evidence indicate that adaptive immunity is also involved in the pathogenesis of AOSD. Increased α-soluble receptors of IL-2 may suggest T-cell activation and proliferation in AOSD patients. Increased IL-4- and IFN-γ-producing T cells were found in active AOSD and related to disease severity. Frequencies of Treg cells in AOSD were significantly lower and were inversely correlated with disease severity. According to these, more and more researchers have reached a consensus that AOSD is a disease at the crossroads of innate immunity and autoimmunity. In this review, we will provide a comprehensive insight into AOSD, describing research progress and the immunological mechanism contribution to the disease. In the meantime, different treatment options and the efficacy and safety of various biologic agents are also discussed. A further understanding of AOSD requires closer cooperation among doctors from different departments, and this review will provide a new idea for diagnosis and therapeutic options.

## Introduction

Adult-onset Still’s disease (AOSD), first reported by Eric Bywaters in the 1970s (1), is a rare disease with unknown etiology that affects multiple systems and organs. Clinical symptoms usually included spiking fever, arthritis, evanescent salmon-pink eruptions, lymphadenopathy, splenomegaly, and other manifestations. Laboratory findings often present as neutrophilic leukocytosis, elevated erythrocyte sedimentation rate (ESR) and C-reactive protein (CRP), hyperferritinemia, and increased inflammatory factors. Notably, ANA, RF, and ENA panels are negative (2, 3). Because of the non-specificity of AOSD clinical presentation and laboratory findings, infection, cancer, connective tissue disease, and other disorders must be ruled out before diagnosis. Due to this and its rarity, research on AOSD remains scarce, and its pathogenesis is still unclear. In the traditional view, AOSD is an auto-inflammatory disease, but recently, research has found that there are autoimmune components that play a role in its pathogenesis, implying that AOSD lies at the crossroads of the innate and adaptive immune systems (2). In this review, we provide a comprehensive insight into AOSD, describing the research progress and the immunological mechanism contribution to the disease. Furthermore, different treatment options and the efficacy and safety of various biologic agents are also considered. A deeper understanding of AOSD requires greater interdepartmental coordination, and this review will provide a new idea for diagnosis and therapeutic options.

## Epidemiology

Epidemiological studies of AOSD are regional and limited, with the populations of these studies being relatively small. Among studies conducted in France, Japan, and Turkey, the incidence of AOSD was found to be between 0.16 and 0.62/100,000 people, independent of ethnicity (4–6). The prevalence rate is estimated to be in the range of 1–34 cases/million people, with two peaks in age distribution at 15–25 and 36–46 years and no obvious gender bias (3). According to recent studies, the incidence and prevalence of AOSD have not changed significantly, regardless of geographical boundaries. However, these studies found that women and people aged 50–59 years were more likely to develop the disease, followed by young adults aged 20–39 years (6–10).

## Clinical and laboratory manifestations

**FIGURE 1:**
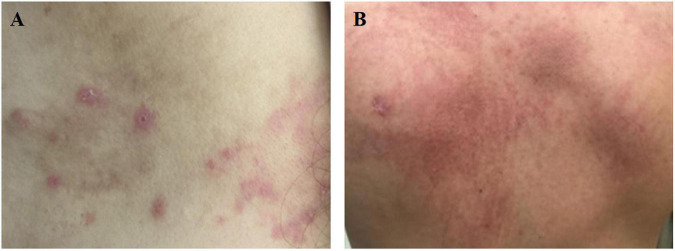
**(A)** Salmon-pink evanescent skin eruptions (ESEs) is which always accompanied by fever. **(B)** Dark red atypical persistent skin eruptions (APSEs), which is usually presented as linear or scratch-like lesions.

Fever is the most common clinical feature of AOSD patients, occurring daily during the patients’ active phase. The onset is sudden, with temperatures often reaching ≥ 39°C and spiking in the afternoon or early evening (2, 3). Moreover, fever can sometimes spontaneously resolve. In some instances, fever can be the only presenting symptom of AOSD, so it is an important consideration when evaluating a fever of unknown origin (2). Arthralgia is known to be the second most common symptom in AOSD, with or without fever (2). All joints of the body may be involved. During early stages, arthralgia is typically mild and transient. Subsequently, joint pain will gradually evolve into chronic arthritis (3). Salmon-pink evanescent skin eruptions (ESEs) accompanied by fever are widely reported in AOSD (2, 3). These kinds of transient and pruritic skin eruptions have been previously considered the most specific clinical manifestation of AOSD (Figure 1A). However, there is an emerging opinion that atypical persistent skin eruptions (APSEs) (Figure 1B) with specific histological manifestations, that is, focal parakeratosis and scattered necrotic keratinocytes in the stratum corneum and the upper one-third of the epidermis, are unique for AOSD, and APSEs might constitute the opposite end of a spectrum that also contains classical ESEs. Some case reports have shown that hematologic or solid malignancies can be secondary to AOSD, with a 9-month median time of diagnosis after initial presentation, so clinicians suggest that APSEs may be an indication of malignancy, thus requiring a closer follow-up (11–13).

In addition, AOSD may present with other symptoms, including pharyngitis, myalgia, lymphadenopathy, hepatomegaly, splenomegaly, pleurisy, and pericarditis (14). According to its clinical course, AOSD can be classically divided into three different patterns: monocyclic, polycyclic, and chronic patterns. About 19–44% of AOSD patients demonstrate the monocyclic pattern, manifesting as self-limiting, systemic symptoms. These patients only have a single flare and achieve complete remission after several weeks or months. Overall, 10–41% of patients demonstrate the polycyclic pattern, where patients have multiple, discrete recurrences systemically or articularly, with remission time between flares; 35–67% of patients exhibit the chronic pattern, with a high frequency of articular symptoms, usually involving wrists, knees, and ankles, which might progress into erosive arthritis (2, 3, 15). There are some rare but life-threatening complications of AOSD like macrophage activation syndrome (MAS), diffuse alveolar hemorrhage, thrombotic thrombocytopenic purpura, diffuse alveolar hemorrhage, pulmonary arterial hypertension, and aseptic meningitis, which should be recognized in the early stage and be managed promptly to decrease morbidity and mortality (16).

With regard to laboratory findings, leukocytosis with an elevated percentage of neutrophils, hyperferritinemia, and increased ESR, CRP, procalcitonin (PCT), and lactic dehydrogenase (LDH) are commonly observed in AOSD; elevated liver enzymes are also reported (2, 3). A newly reported marker is glycosylated ferritin (GF), which usually decreases to the range of 20–50% under inflammatory conditions. In AOSD, the level of GF is remarkably decreased (< 20%) and should be considered to have utility in AOSD diagnosis (17). The distinct advantage of these criteria is that the diagnosis is not of exclusion (2); however, one pitfall is that testing for GF may not be common in most hospitals.

## Etiology and pathogenesis mechanisms

**FIGURE 2:**
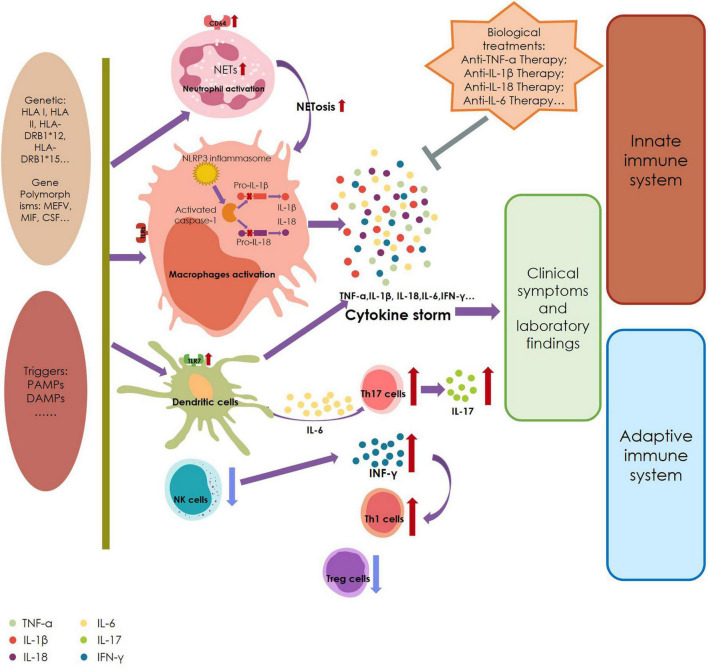
A summary of the pathogenesis of adult-onset Still’s disease. HLA, human leukocyte antigen; MIF, migration inhibitory factor; CSF, colony-stimulating factor; PAMPs, pathogen-associated molecular patterns; DAMPs, damage-associated molecular patterns; TLR, Toll-like receptors; NETs, neutrophil extracellular traps; NLRP3, NLR3-containing a pyrin domain; NK cells, nature killer cells; IL, interleukin; TNF, tumor necrosis factor; INF, interferon.

At present, the etiology of AOSD is still unclear, but researchers believe that its pathogenesis may be related to infection, genetics, and abnormal immune function (Figure 2).

### Genetics

Combined with the epidemiological studies mentioned earlier, current reports of AOSD have not shown obvious familial trends. AOSD occurs in different regions and ethnic groups without significant restriction, but there is still a certain genetic background. Several studies have shown that genes associated with AOSD are mainly human leukocyte antigen class I and class II (HLA I, HLA II), such as HLA-B17, -B18, -B35, -DR2, -DR4, -DR7, and -Bw35 (18–20). Some studies have found that the incidence of AOSD is more strongly related to HLA-DRB1*12 and -DRB1*15, while another study found that the incidence of AOSD is negatively correlated with HLA-DR1 and HLA-DRB1*04 (20, 21). Variations in the MEFV gene have been found to be associated with AOSD in Korea, Japan, Germany, and Turkey (22–25). Scholars in Turkey have also found that mutations in the TNFRSF1A gene are also associated with AOSD, but larger samples are still needed for verification (25).

Gene polymorphism is one of the research hot spots of AOSD. In 2002, N Kamatani et al. found that genetic polymorphisms in human IL-18 are associated with susceptibility to AOSD (26). Chen et al. found a functional association between IL-18 gene-607 (C/A) promoter polymorphisms and disease course in Chinese patients with AOSD, and this genotype with a low IL-18 level could be a protective factor against both AOSD severity and progression to chronic disabling arthritis (27). Functional promoter polymorphisms in the migration inhibitory factor (MIF) gene have been reported to influence plasma MIF levels in AOSD and might be associated with disease susceptibility or clinical presentation (28). Another study found that SNP rs11102024, which is 5′ upstream of colony-stimulating factor 1 (CSF1), was associated with AOSD; this gene encoding macrophage-CSF (M-CSF) and the rs11102024 T allele were related to higher M-CSF levels and a systemic rather than chronic articular manifestation of AOSD (29). Chen et al. found that NLR family pyrin domain containing 3 (NLRP3)-inflammasome signaling was strongly associated with AOSD; they studied single-nucleotide polymorphisms (SNPs) involved in NLRP3-inflammasome signaling and reported that SNP rs11672725 of the CARD8 gene was significantly associated with AOSD susceptibility. Patients with the rs11672725CC genotype were more likely to have both low CARD8 levels and a systemic pattern of disease (30). Hung, Chen and Lan et al. reported another update last year showing that SNPs of autophagy-related 16-like 1 (ATG16L1) gene have associations with the AOSD clinical phenotype in particular, and the AA/CC/TT haplotype of this gene might be linked to the systemic pattern of manifestation and specific clinical features of AOSD, that is, skin rash (31). However, these results are still awaiting demonstration in a larger, more diverse population.

### Infections

The main clinical manifestations of AOSD are fever and sore throat. Laboratory indices often find an elevated C-reactive protein (CRP), erythrocyte sedimentation rate (ESR), and leukocytosis with neutrophilia, which are similar to findings in infectious diseases. Therefore, scholars believe that AOSD pathogenesis is likely related to bacterial or viral infection. There are many reports of AOSD secondary to bacterial or viral infection, including EB virus; cytomegalovirus; coxsackie virus; parvovirus B19; influenza virus; parainfluenza virus; adenovirus; hepatitis A, B, and C viruses; human immunodeficiency virus; rubella virus; *Mycoplasma pneumoniae*; *Chlamydia pneumoniae*; *Streptococcus pneumoniae*; *Yersinia enterocolitica*, *Campylobacter jejuni*, and *Borrelia burgdorferi* (2, 3, 32). As for whether these pathogens are directly pathogenic or participate in the development of AOSD as a trigger, there is still controversy. Most authorities believe that infection only plays a trigger role.

### Innate immune system

According to its clinical manifestations, AOSD has distinct features of an autoinflammatory disease; thus, the innate immune system is heavily involved in its pathogenesis. External factors like environmental factors and infectious triggers could activate innate immune cells *via* Toll-like receptors (TLRs), leading to an abnormal response of the innate immune system and even cytokine storm. Overproduced cytokines like IL-1 could then trigger adaptive immune cells, such as Th17 cell-mediated response.

TLRs are expressed on various cells, such as dendritic cells (DCs), neutrophils, natural killer (NK) cells, macrophages, mast cells, B cells, T cells, and other non-immune cells (33). The major function of TLRs is to sense and transfer danger signals, damage-associated molecular patterns (DAMPs), and pathogen-associated molecular patterns (PAMPs) to intracellular signaling pathways (34). TLRs also help in the recruitment of neutrophils, activation of inflammation, and amplification of Th-17-driven inflammatory responses (35). In AOSD patients, serum S100 A8/A9 and A12 have been reported to be elevated (36–38). TLR4 and receptor of advanced glycation end products (RAGEs) signaling could be activated by S100 A8/A9 and A12, indicating the overproduction of pro-inflammatory cytokines, and then inflammatory cells like neutrophils and monocytes are activated. The expression of TLR7 was significantly increased in dendritic cells of AOSD patients compared to healthy controls and was concurrent with elevated transcript and protein levels of MyD88, IRAK4, and TRAF6, indicating activation of the TLR7-MyD88 pathway. The expression level of TLR7 was positively correlated with both serum levels of cytokines (IFN-γ, IL-1β, IL-6, and IL-18) and disease activity (35, 38, 39), and it could be effectively relieved after aggressive treatment.

Neutrophils are strongly activated in the pathogenesis of AOSD, and neutrophilia is seen in over 80% AOSD patients (14). CD64, also known as Fc gamma-receptor I (FcγRI), is upregulated in neutrophils in AOSD patients, suggesting that neutrophils are activated (40). Triggering receptor expressed on myeloid cells-1 (TREM-1) is a major amplifier of inflammatory signaling, and the soluble form of TREM-1 (sTREM-1) was found to be elevated in the serum of AOSD patients, with direct correlation to disease activity (41). However, the function of sTREM-1 is still unclear, but it has been postulated that sTREM-1 weakens the TREM-1-mediated inflammatory response and negatively regulates TREM-1 signaling (42).

Neutrophil extracellular traps (NETs) have been extensively studied in AOSD recently. NET is a way of neutrophil death, and the release of NETs is known as an effector mechanism of polymorphonuclear neutrophils (43). Several studies revealed that NETs are significantly elevated in AOSD patients (43–46). In addition, neutrophils in AOSD patients have a greater capability to form NETs, which then leads to the activation of pro-inflammatory macrophages and the NLRP3 inflammasome. Moreover, the formation of NETs plays a linking role between neutrophils and macrophages in the occurrence of cytokine storm (47). S100 A8/A9 and A12 are parts of NETs, function as DAMPs, and act as the ligand of TLR4 or RAGE, involved in the pathogenesis of AOSD by enhancing the feedback loop (36, 48, 49). IL-18 is widely reported to be increased in the serum of AOSD patients, and an updated study found that it regulates mitochondrial ROS generation by increasing calcium influx to induce the formation of NETs in a process which could be suppressed by microRNA-223 (50). Hu and colleagues reported a gene of leukocyte immunoglobulin-like receptor (LIR)-A3 (LILRA3) that plays a pathogenic role in AOSD and speculated that the functional LILRA3 is a new genetic susceptibility factor for neutrophil activation and NETosis in AOSD (51). They conducted a study that revealed increased type I interferon (IFN) could induce NETs, especially ones containing oxidized mitochondrial DNA in AOSD (46). IL-6-producing low-density granulocytes (LDGs) are also elevated in active-stage AOSD patients and correlate with particular clinical manifestations (44, 52).

Activation of macrophages is another hallmark of AOSD. After recognition of DAMPs and PAMPs, TLRs on macrophages recruit adapter molecules like MyD88 to activate the downstream cascade signaling pathway by NF-κB, thereby driving pro-inflammatory cytokine storms (53–55). The engaged NLRP3 inflammasome activatescaspase-1, which then converts pro-IL-1β and pro-IL-18 into mature IL-1β and IL-18, join with various cytokines from other pathways, for example, IL-6, IL-8, IL-10, TNF-α, and IFN-γ, to amplify the inflammatory response in a cytokine burst that leads to the onset of AOSD (2, 54–56). Markers of macrophage activation, e.g. macrophage migration inhibitory factor (MIF) (57, 58), a proinflammatory cytokine with the ability of upregulating the expression of proinflammatory mediators, and macrophage-colony stimulating factor (M-CSF) (59) were reported to be increased in the serum of AOSD patients and correlated with disease activity. The heme receptor expressed on macrophages, sCD163, is found to be elevated and related to hyperferritinemia in AOSD (60). Hyperferritinemia is commonly seen in AOSD patients, and ferritin is considered to be an important mediator of AOSD (56). The release of ferritin is stimulated by activated macrophages, and both ferritin in the circulation and that infiltrated into the skin are correlated with the severity of AOSD (56, 61). Although there remains a paucity of research on macrophages in AOSD, existing evidence shows that macrophages play a vital role in the pathogenesis and clinical implications of AOSD.

NK cells are also important in the innate immune system and function by secreting cytolytic granules like granzyme and perforin and inducing caspase-dependent apoptosis through pathways like Fas-Fas ligand (62). In AOSD, NK cell abundance and cytotoxic function were found to be significantly decreased compared to healthy controls (63, 64), and this dysfunction might lead to excessive activation of macrophages and T cells, favoring the development of AOSD (62, 65).

### Adaptive immune system

Since the early 2000s, ever-increasing evidence has indicated that the adaptive immunity is also involved in the pathogenesis of AOSD. In 2003, a serum cytokine study of AOSD found increased concentrations of CD25, the α-soluble receptor of IL-2, possibly indicating the activation and proliferation of CD4 + T cells in AOSD (66, 67). A year later, Japanese scientists reported their belief that AOSD reflects a Th2, rather than a Th1, cytokine profile, finding high levels of IL-4 and IL-13 but normal values for INF-γ, IL-12, and IL-2in AOSD patients (68). The elevation of IL-4 and the infiltration of IL-4-producing T cells in the skin, sera, and synovial tissues of AOSD patients might reflect the Th1 polarization of CD4 + T cells (3, 67). Increased IL-4-producing T cells and IFN-γ were found in active AOSD patients and related to disease severity (3, 69). This predominance of Th1 response in AOSD likely causes activation of NK cells and macrophages and promotes cell-mediated immunity (35, 69). IL-6, IL-1β, and IL-18 are elevated in AOSD and involved in its pathogenesis (67, 69–71). Researchers found that IL-6 and IL-1β have the ability to induce naive T-cell differentiation into Th17 cells (72, 73) and that IL-18 synergizes with IL-23 to generate IL-17-producing CD4 T cells (73, 74). Increased numbers of circulating Th17 cells and levels of serum Th17-related cytokines are observed in AOSD and are strongly associated with disease activity (73). Circulating CD4^+^CD25*^high^* regulatory T cells (Treg), an anti-immune mechanism, and TGF-β are found to be diminished in AOSD, inversely correlated with disease severity in AOSD, suggesting that deficiency of Tregs leads to dysfunction of the immune system in AOSD patients (75). Another study found that in Tregs of AOSD patients, the expression levels of IFN-γ, IL-17, and IL-4 were significantly increased, but the expression level of FoxP3 was significantly decreased. It also illustrated the suppression function of Tregs in AOSD was impaired (76). In summary, recent studies have reached the consensus that AOSD is a disease at the crossroads of innate immunity and autoimmunity.

### Cytokines

Dysregulation of pro-inflammatory cytokines plays a vital role in mediating the pathogenesis of AOSD, and its serum cytokine profile has been well studied, especially in the context of overexpression of TNF-α, IL-1β, IL-6, IL-18, and IFN-γ (Table 1).

**TABLE 1:** Cytokine profile and targeting symptoms.

Cytokine	Targeting symptoms (3)
IL-1β	Systemic inflammation, cartilage destruction and bone destruction
IL-18	Systemic inflammation, liver involvement, and MAS
IL-6	Fever, skin eruption, joint manifestations and disease activity
TNF-α	Systemic inflammation

TNF-α is a classic pro-inflammatory cytokine largely produced by macrophages and lymphocytes (77). Synoviocyte proliferation, osteoblastosis, and cachexia are reported to be associated with TNF-α (35). As mentioned previously, macrophages are strongly activated by DAMPs and PAMPs, which might lead to the elevation of TNF-α in the serum of AOSD patients. In the amplification phase of AOSD, cytokine storm occurs following a caspase cascade, causing different cytokines to interact and promote each other, subsequently resulting in a large release of pro-inflammatory cytokines that causes tissue damage. One study reported that TNF-α levels in synovial membranes of AOSD patients show a significantly increase compared to healthy controls, suggesting that the overexpression of TNF-α might lead to arthritis (69).

IL-1β, a highly studied member of the IL-1 family, is a pro-inflammatory cytokine produced and secreted by innate immune cells like macrophages and monocytes (78). It is synthesized as an inactive 31-kDa precursor peptide, that is pro-IL-1β, and is cleaved by pro-inflammatory protease caspase-1 into its 17-kDa mature form, known as mIL-1β (79). Under the stimulation of DAMPs and PAMPs, inflammasomes, for example, NLRP3, in innate immune cells are recruited and subsequently activate caspase-1 to convert inactive pro-IL-1β into the active mature form, and the mature IL-1β is rapidly secreted from cells to exert inflammatory effects by binding to the IL-1 receptor type 1 (IL-1R1) (80, 81). The expression levels of NLRP3, caspase-1, and IL-1β are all significantly increased in AOSD patients (47, 67, 82). However, following treatment targeted against NLRP3, protein expression of NLRP3 and IL-1β, as well as IL-18, in PBMC significantly decreased in patients with AOSD (82). This suggests that the activation of NLRP3, caspase-1, and IL-1β is indispensable in the pathogenesis of AOSD. Moreover, the RNA level of IL-1β in skin eruptions of AOSD patients is significantly increased compared to that in healthy controls (83). Although there is no statistically differentiation between the RNA level of IL-1β in APSEs and ESEs, IL-1β is strongly expressed in the upper third of the epidermis of APSEs, which might be correlated with the formation of APSEs (13).

IL-18 is also one of 11 members of the IL-1 family. It is first synthesized as an inactive precursor with a molecular weight of 24,000 and subsequently cleaved by caspase-1 into its active mature form with a weight of 17,200. Much like IL-1β, inactive procaspase-1 is converted into active caspase-1 by the inflammasome, that is, NLRP3, and subsequently, pro-IL-18 is cleaved into mature IL-18 and secreted from macrophages or monocytes (84). The secreted IL-18 binds to IL-18 alpha chain (IL-18Rα) and the co-receptor, IL-18 receptor beta chain (IL-18Rβ), to form a heterodimeric signaling complex. Following this, Toll-IL-1 receptor (TIR) domains trigger the heterodimer binding to MyD88, subsequently inducing phosphorylation of four IRAKs, TRAF-6, and activation of NFκB (84, 85). The main immunoregulating function of IL-18 is inducing IFN-γ from NK cells (84). IL-18-binding protein (IL-18BP) is a secreted protein with high affinity for IL-18 (400 pM), which is significantly higher than that for IL-18Rα, and it can reduce the induction of IFN-γ by binding to IL-18, thereby downregulating Th1 responses (86). In AOSD, IL-18 is elevated in the serum, lymph node, skin eruptions, and synovial membranes (67, 69, 87, 88), and the level of IL-18 is correlated with serum ferritin values, hepatitis severity, disease severity, and disease activity (69, 70, 89, 90). Several studies have also detailed the possibility of IL-18 as a diagnostic biomarker for differentiating AOSD from sepsis and normal health (89, 91, 92).

The elevation of IL-6 in the serum of AOSD patients, correlating with disease activity, has been widely reported (67, 69, 93, 94), and recently, studies have also found its overexpression in skin eruptions of AOSD (83). IL-6 is a well-known pleiotropic cytokine involved in both pro- and anti-inflammatory processes. In the early stage of inflammation, IL-6 is synthesized in a local lesion and then moves to the liver through the bloodstream. Subsequently, acute-phase proteins, such as C-reactive protein (CRP) and serum amyloid A (SAA), are induced rapidly, which might contribute to the abnormal laboratory results observed in AOSD (93, 95, 96). Like the pro-inflammatory cytokines mentioned before, the transcription of the mRNA of IL-6 could be enhanced by activated NF-κB signaling after the stimulation of DAMPs and PAMPs. TNF-α and IL-1β also have the ability to activate transcription factors to produce IL-6 (96). According to existing studies, IL-6 is reported to have an impact on the function of osteoclasts and synovial tissues (97–99). Together, these findings might suggest that IL-6 could be responsible for clinical features like fever and arthritis (67, 69, 93, 100).

The function of IFN-γ in AOSD is both controversial and unclear. The serum level of IFN-γ and expression of IFN-γ in CD4 + cells are elevated and correlated with disease activity in AOSD (101). Although the proportion of NK cells is decreased in AOSD, their ability to produce IFN-γ is higher than that in healthy people, suggesting that IFN-γ is strongly involved in the inflammatory process (64, 101). Some experts believe that IFN-γ functions by inducing overexpression of cytokines or chemokines like IL-18 and CXCL-10 in the inflammatory pathogenesis of AOSD; moreover, these induced cytokines directly correlate to disease activity, so they can be used as biomarkers of disease activity and treatment efficacy (102–104). Also, in skin eruptions, IFN-γ is expressed at a high level, especially in the upper third of the epidermis (13, 83). This peculiar expression pattern might suggest that IFN-γ contributes to the formation of the specific pathological phenomenon of APSEs.

Other cytokines like IL-10 and IL-37 are reported to be elevated in the serum of AOSD patients and associated with disease activity (105, 106). Mimura et al. found that IL-10 has dual roles in stimulating NK cells and inhibiting monocytes, which might contribute to AOSD (107). Both IL-17 levels and Th17 cell numbers were significantly high in AOSD patients and correlated with disease activity, which suggests that treatment targeting Th17 cells and IL-17 could play a potential therapeutic role in AOSD (73).

## Diagnosis

Because there is a lack of specific clinical symptoms and laboratory markers, the diagnosis of AOSD remains problematic for clinicians. Only two sets of diagnostic criteria have been verified by research and clinical studies (Table 2). The most widely used criteria are those published in 1992 by Yamaguchi et al. (108), with a sensitivity of 96.3%, specificity of 98.2%, positive predictive value (PPV) of 94.6%, and negative predictive value (NPV) of 99.3% (2, 109). However, the drawback is that infection, malignant tumors, and other connective tissue diseases should be carefully excluded when using these criteria. The other is the Fautrel criteria, with a sensitivity of 87.0%, specificity of 97.8%, PPV of 88.7%, and NPV of 97.5% (2, 109).

**TABLE 2:** Diagnostic criteria of AOSD.

Yamaguchi criteria	Fautrel criteria
**Major criteria**	
Arthralgia lasting 2 weeks or more	Arthralgia
Fever ≥ 39°C lasting 1 week or more	Spiking fever ≥ 39°C
Typical skin rash: maculopapular, non-pruritic, salmon-pink rash with concomitant fever spikes	Transient erythema
WBC ≥ 15 × 109 (N%>80%)	Pharyngitis; Neutrophil polymorphonuclear proportion (PMN) ≥ 80%; GF proportion ≤ 20%
**Minor criteria**	
Pharyngitis or sore throat	Typical rash
Lymphadenopathy and/or splenomegaly	WBC ≥ 10 × 109
Liver enzyme abnormalities (aminotransferases)	
ANA and RF:(-)	
**Diagnosis**	
At least five criteria, including two major criteria and no exclusion criteria (Absence of infection, malignant diseases, inflammatory disease)	Four major criteria or three major criteria and two minor criteria

## Treatment

**FIGURE 3:**
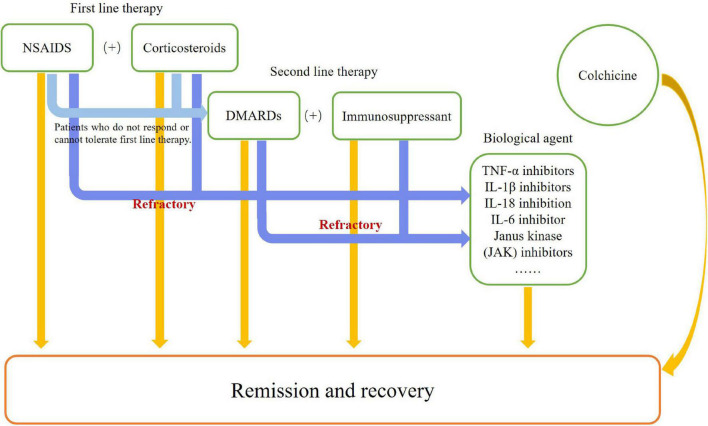
Flowchart of therapies.

Because the pathogenic mechanism of AOSD remains unclear, other diseases need to be excluded before establishing a diagnosis. In addition, its treatment is mostly empirical and based on sporadic cases, retrospective cases studies and small clinical trials (Figure 3). The main purpose of its treatment is to address symptoms and control inflammation. Some scientists emphasize the importance of taking disease phase, primary clinical symptoms, and probable complications into consideration before identifying an appropriate therapeutic strategy for individual AOSD patients (35, 110). The treatment of AOSD can be generally summarized into two parts: traditional treatment and biological treatment.

### Traditional therapies

The classical traditional therapies usually include non-steroidal anti-inflammatory drugs (NSAIDs), corticosteroids, and disease-modifying antirheumatic drugs (DMARDs). NSAIDs and corticosteroids are considered first-line therapy for AOSD (3, 14). Typically, NSAIDs are used in mild to moderate patients, with no more than 20% of cases achieving remission of fever and musculoskeletal symptoms (14, 35). Notably, careless use of NSAIDS can lead to severe side effects, for example, high doses of indomethacin and aspirin can precipitate fulminant hepatic failure when the transaminase level is high (14). Corticosteroids are widely used to suppress inflammatory responses, and about 60–70% of patients have effective response to this therapy, especially those with systemic symptoms. Clinically, corticosteroids usually begin at a dosage of 0.5 mg-1 mg/kg/day, but an initial dosage of at least 0.8 mg/kg/day of prednisone might result in quicker resolution of symptoms with less relapse than with the usage of lower doses (111, 112). If severe complications like macrophage activation syndrome (MAS) occurs, intravenous high-dose corticosteroids should be taken into consideration. After remission of clinical symptoms and return to normal laboratory parameters, the dosage of corticosteroids should be tapered slowly. However, there are some side effects, with about 40–45% patients developing steroid dependency; Cushing syndrome, osteoporosis, and aseptic osteonecrosis have also been reported (111, 113).

DMARDs, such as methotrexate (MTX), cyclosporin A (CsA), and leflunomide, are second-line therapy for AOSD in patients who do not respond or cannot tolerate first-line therapy. MTX is the most common DMARD used in this disease. A retrospective study of 26 AOSD patients showed that under the dosage of 7.5–17.5 mg/week, 23 patients responded to MTX, with no difference between patients with or without extra-articular symptoms, and 18 of them achieved complete remission (114). However, MTX is hepatotoxic, and liver function must be monitored during its use.

### Colchicine

Colchicine is an ancient, inexpensive, and readily available anti-inflammatory drug widely used in autoinflammatory diseases like gouty arthritis and familial Mediterranean fever (FMF). It functions by impairing neutrophil recruitment and suppressing the NLRP3 inflammasome and caspase-1 activation (115–117). Recently, increasing numbers of clinical doctors have found that using colchicine alone or in combination shows high efficacy in the treatment of refractory AOSD. Shi and Rao reported an AOSD case accompanied by pulmonary tuberculosis that was successfully treated with a 2-month regimen of 0.5 mg/bid per day of colchicine, resulting in disease resolution without recurrence (118). AOSD patients with mutations of the MEFV gene have also been reported to be responsive to colchicine therapy (119). Moreover, colchicine in combination with NSAIDs and immunosuppressive agents has shown excellent efficacy also (120, 121).

### TNF-α inhibitors

At present, infliximab, etanercept, and adalimumab are the most common TNF-α inhibitors in the clinic. The usage of this class of drugs in AOSD treatment can be tracked back to the early 2000s. In 2001, Manger et al. reported a study of infliximab usage in six AOSD patients. The results showed that after patients received 3–5 mg/kg infliximab on weeks 0, 2, and 6, clinical symptoms like fever, arthralgias, skin rashes, myalgias, and hepatosplenomegaly resolved in all patients. After three courses of infliximab treatment, all clinical manifestation and laboratory indicators were found to be normal and stable (122). A year later, a report of etanercept in the treatment of AOSD was published. Researchers reported 12 AOSD patients who had active arthritis and were resistant to DMARDs, 25 mg etanercept biweekly for 6 months. Except for two cases of withdrawal due to disease flare-up, all other patients obtained satisfactory results with no adverse events. Arthritis in five of 10 patients showed improvement (123). In 2004, Fautrel et al. conducted a study to assess the efficacy and tolerability of TNF-α inhibitors in refractory AOSD. Under treatment using infliximab and etanercept, only five of 25 cases achieved complete remission: one receiving etanercept and four receiving infliximab (124). However, one case report found that the increased TNF-α level due to etanercept administration might lead to an exacerbation of AOSD (125). The earliest reported use of adalimumab was in 2005 (126). Overall, TNF-α inhibitors appear well tolerated in AOSD treatment. They show a better efficacy in treating the chronic articular pattern, but when considering suppression of systemic manifestations, they have been less efficacious than IL-1 and IL-6 inhibitors.

### IL-1β inhibitors

As IL-1β is a key pro-inflammatory cytokine in the immunopathogenesis of AOSD, many believe that targeted therapy on this cytokine has highest likelihood of achieving a therapeutic effect. IL-1β inhibitors are the most widely used biological agents in the treatment of AOSD and are supported by a series of clinical trials. Anakinra (ANK), canakinumab (CNK), and rilonacept are currently the most commonly available biologics used in AOSD.

Anakinra is a recombinant form of the human IL-1 receptor (IL-1R) antagonist which blocks biological activity of both IL-1β and IL-1α. It is widely used in auto-inflammatory diseases, and the earliest report of ANK usage in refractory AOSD can be traced back to 2005 (127). In 2007, clinicians from France assessed efficacy and the safety of ANK (100 mg per day) treatment in 15 AOSD patients. Clinical features and laboratory indicators in nine of 15 patients showed at least 50% improvement at 6 months, and a total of 11 patients responded to ANK (128). A case study with 25 AOSD patients found that after subcutaneous injections of ANK (100 mg/day), 84% of patients showed complete clinical remission within a few days. Moreover, 82% of patients achieved an American College of Rheumatology 20 (ACR20) score at 1 month, and this increased to 100% at 1 year (129). The first multicenter study of ANK in AOSD was conducted in 2012 with 22 patients, and their results showed that ANK produced better efficacy than DMARDs (130). Another multicenter study in 41 AOSD patients showed that ANK yielded rapid and sustained clinical and laboratory improvement in refractory AOSD patients who did not response to traditional therapies, but arthritis was found to be more refractory than systemic symptoms (131). A large multicenter retrospective study with 140 AOSD patients also strongly demonstrated the prominent efficacy of ANK in refractory AOSD, with significant improvement of patients’ clinical symptoms and Pouchot’s scores (132). After 2 years, an Italian study assessed the drug retention rate (DRR) of ANK in AOSD under a long-term follow-up and found that the DRR was excellent with 44.6% retained at the 60th month and 30.5% at the 120th month. It is worth noting that the concomitant use of DMARDs did not affect the DRR of ANK. The number of swollen joints at the baseline of therapy was positively related to the risk of losing ANK efficacy, and skin rash was a negative predictor of sustained remission-associated ANK withdrawal (133). In the same year, a panel of Italian experts established 11 evidence- and consensus-based statements, including that it is relatively safe and effective to use anti-IL-1 therapies in refractory AOSD patients, especially those with the systemic pattern, and it could be both first and subsequent lines of treatment using biologic agents (134). An updated study reported that patients could attain better remission in systemic inflammation and joint manifestation if they were treated with IL-1 inhibitors as soon as possible after disease onset (135).

Canakinumab is an IL-1β long-acting inhibitor. Due to its half-life of 26 days, patients can receive 150 mg CNK every 4–8 weeks. In 2016, CNK was officially approved by the European Medicines Agency (EMA) and the United States Food and Drug Administration (FDA) for the treatment of systemic juvenile idiopathic arthritis (sJIA) and AOSD (35). CNK is not as widely used as ANK, and the first report of CNK was published in 2012, with successful application in two refractory AOSD cases, which had demonstrated resistance to short-acting IL-1 blockade (136). Although it failed to reach its primary endpoint, a randomized, multicenter phase II clinical trial in 2020 showed that 4 mg/kg of CNK, with a maximum of 300 mg subcutaneous every 4 weeks, had the ability to improve several laboratory markers of patient outcomes in AOSD (137). Of nine AOSD patients treated with CNK for 15.00 ± 12.3 months, eight achieved clinical remission at 3-month assessment. The daily dosage of corticosteroids was reduced during the follow-up, and no severe adverse events were seen (138). Another retrospective multicenter study showed that about 78% of refractory AOSD patients achieved complete clinical symptom resolution and laboratory parameter normalization within 3 months of CNK therapy (139). Nevertheless, the safety and efficacy of CNK remain to be explored in larger, multinational AOSD cohorts in future.

Rilonacept, a soluble IL-1 trap molecule, binds to both IL-1α and IL-1β with high affinity. The data on rilonacept are limited, with a case report in 2012 showing that it could alleviate both systemic and arthritic symptoms in AOSD (140).

### IL-18 inhibition

IL-18 inhibitor used in AOSD has been tadekinig alfa (recombinant IL-18BP). In 2018, an open-label, multicenter, phase II clinical trial was the first of its kind to examine the safety and efficacy of IL-18 blockade in AOSD treatment. After using doses of 80 mg or 160 mg of tadekinig alfa three times per week, nearly 50% of patients achieved remission of fever and > 50% reduction of CRP values from baseline after 3 weeks (141). Another study reported rapid and dramatic reduction of serum IL-18 and clinical symptoms along with correction of laboratory test abnormalities following therapy using tadekinig alfa, suggesting that IL-18 blockade may be used to achieve a therapeutic effect in AOSD (142). Although the data are limited, IL-18 and IL-18BP are still potent biological therapeutic target for AOSD, more national-wide multicenter clinical trials with larger population sizes are needed in future.

### IL-6 inhibitor

Tocilizumab (TCZ), also known as myeloma receptor antibody (MRA), is a humanized anti-human IL-6R monoclonal antibody first used for treatment of refractory AOSD in 2002 (143). TCZ has a relatively long half-life and can be administered intravenously biweekly or monthly. Early case reports of the usage on TCZ in AOSD found that both systemic and joint symptoms improved and that inflammatory indicators like CRP, SAA, and ESR normalized rapidly (143–146). In 2011, Puéchal et al. treated 14 refractory AOSD patients with TCZ (5–8 mg/kg every 2 or 4 weeks). A total of 11 of them successfully completed the 6-month treatment; results showed that 36% patients achieved remission at 3 months and 57% at 6 months (147). In a retrospective multicenter study of 34 AOSD patients, 22 of them intravenously received TCZ with initial dosages of 8 mg/kg every 4 weeks, two patients received 4 mg/kg every 4 weeks, and 10 patients received 8 mg/kg every 2 weeks. Both clinical symptoms and laboratory parameters improved rapidly, and after 1 year of treatment, the incidence of arthritis, skin rashes, fever, and lymphadenopathy had reduced dramatically. This was accompanied by a similar extreme decrease in inflammatory indicators and dosage of prednisone (148). Another multicenter retrospective study of 22 cases reported in Korea showed that 50% of patients achieved a decrease of > 2 in the modified Pouchot’s scores of the study at 6 months and 64.3% at 12 months (149). Ma et al. conducted a meta-analysis on the efficacy and safety of TCZ in AOSD; partial and complete remission rates were calculated to be 85.38% and 77.91%, respectively. The remission rate of refractory AOSD patients after TCZ treatment was 87.92% (150). In total, these studies emphasized that clinical manifestations and laboratory parameters of AOSD can be improved by treatment using TCZ, and the need for corticosteroids could be reduced. In summary, treatment with TCZ is effective, safe, and well tolerated for AOSD.

### Other treatments

Baricitinib and tofacitinib, Janus kinase (JAK) inhibitors, have also seen use in AOSD treatment (151, 152), but their efficacy and safety should be verified with more studies. The successful use of rituximab, a monoclonal anti-CD20 antibody that targets B cells, in refractory AOSD patients has been reported (153–155). Intravenous immunoglobulin (IvIg) was also reported to be an effective and well-tolerated therapy in AOSD patients (156).

## Conclusion

Due to the non-specificity of symptoms and laboratory parameters in AOSD, the diagnosis and treatment of the disease usually require multidisciplinary collaboration. The rarity of AOSD has limited the discovery of new treatments and prevented the full understanding of its pathogenesis. In this review, we detailed the immunopathogenic mechanism of AOSD and current novel therapies by summarizing existing references, with the aim to unravel the mysteries of this disease, which stand at the crossroads of innate immunity and autoimmunity. Future studies in the autoimmune aspect of AOSD and on novel biological agents acting on different targets are still needed.

## Author contributions

SR is responsible for drafting this manuscript. LT is responsible for revising the language of this manuscript. MZ is responsible for revising this manuscript. WS and QL contributed to the final approval of the article. All authors contributed to the article and approved the submitted version.
